# Whole transcriptome analysis of *Penicillium digitatum* strains treatmented with prochloraz reveals their drug-resistant mechanisms

**DOI:** 10.1186/s12864-015-2043-x

**Published:** 2015-10-24

**Authors:** Jing Liu, Shengqiang Wang, Tingting Qin, Na Li, Yuhui Niu, Dandan Li, Yongze Yuan, Hui Geng, Li Xiong, Deli Liu

**Affiliations:** Hubei Key Laboratory of Genetic Regulation and Integrative Biology, School of Life Science, Central China Normal University, Wuhan, 430079 China

**Keywords:** Transcriptome, *Penicillium digitatum*, Gene expression, Prochloraz response, Prochloraz resistance

## Abstract

**Background:**

*Penicillium digitatum* is one of the most destructive postharvest pathogen of citrus fruits, causing fruit decay and economic loss. The emergence of fungicide-resistant strains made the control of *P. digitatum* more difficult. While the genome of *P. digitatum* is available, there has been few reports about its resistant mechanism from the transcriptome perspective and there has been no large-scale functional annotation of the genome using expressed genes derived from transcriptomes.

**Methods:**

Total RNA of* P. digitatum *strain HS-F6 (prochloraz-resistant strain) and HS-E3 (prochloraz-susceptible strain) before and after prochloraz-treatment were extracted and sequenced on an Illumina Hiseq 2000 platform. The transcriptome data of four samples were compared and analyzed using differential expression analysis, novel transcripts prediction and alternative splicing analysis, SNP analysis and quantitative real-time PCR.

**Results:**

We present a large scale analysis about the transcriptome data of *P. digitatum*. The whole RNA was extracted from a prochloraz-resistant strain (HS-F6) and a prochloraz-susceptible strain (HS-E3) before and after prochloraz-treatment and sequenced by Illumina technology. A total of more than 100 million reads were generated and de novo assembled into 9760 transcripts that contained annotated genes after quality control and sequence assembling. 6625 single nucleotide variations (SNVs) were identified from the sequences aligned against the reference genome. Gene expression profiling analysis was performed upon prochloraz treatment in HS-F6 and HS-E3, and differential expression analysis was used to identify genes related to prochloraz-response and drug-resistance: there are 224 differentially expressed genes in HS-E3 and 1100 differentially expressed genes in HS-F6 after prochloraz-treatment. Moreover, gene expression profile in prochloraz-resistant strain HS-F6 is quite different from that in HS-E3 before prochloraz-treatment, 1520 differential expression genes were identified between the two strains. Gene ontology (GO) term enrichment and KEGG enrichment were then performed to classify the differential expression genes. Among these genes, there are a lot of transporter encoding genes including 14 MFS (Major Facilitator Superfamily) transporters, 8 ABC (ATP-binding cassette transporter) and 3 MATE (multidrug and toxic compound extrusion family) transporters. Meanwhile, the roles of typical MFS, ABC and MATE proteins in prochloraz resistance were investigated using real-time quantitative PCR.

**Conclusions:**

The sequencing-based transcriptome data of *P. digitatum* demonstrate differences between prochloraz-resistant and prochloraz-susceptible strains with prochloraz-treatment. The differences existed in expressed transcripts, splice isoforms and GO categories, which would contribute to our knowledge on the molecular mechanisms involved in drug resistance of *P. digitatum*.

**Electronic supplementary material:**

The online version of this article (doi:10.1186/s12864-015-2043-x) contains supplementary material, which is available to authorized users.

## Background

Ascomycete fungi *P. digitatum* is the most destructive disease of citrus fruit which cause green mold and responsible for up to 90 % of total crop losses during postharvest packing, storage, transportation, and marketing [1]. Control of *P. digitatum* is becoming more difficult because of the emergence of drug-resistant strains due to excessive use of demethylation inhibitor (DMI) fungicides [2–4].

Fungal resistance to azole reagents has been attributed to various genetic mutations in its target gene *erg11* (*cyp51*), and/or the upregulation of efflux pump genes such as *MDR1*, *CDR1,* and *CDR2* [5]. Transcription factors acting on effector genes is another important attribution to drug resistance(s) characterized in a number of clinical species [6]. For example, CaUpc2 is a well-characterized transcription factor in *Candida albicans* that is related with drug resistance and sterol metabolism [7, 8]. Sterol regulatory element-binding proteins (SREBPs) contain a basic helix-loop-helix domain with a specific tyrosine residue and function as membrane-bound transcription factors required for virulence, resistance to antifungal drugs, and hypoxia responses in fungi [9]. In 2015, we reported the identification and characterization of an SREBP protein, SreA, in *P. digitatum* and proved that it played an important role in the full virulence, prochloraz (PRC) resistance and expression of ergosterol biosynthetic genes in *P. digitatum* [10]*.* Evidence on the transcriptional regulation of these target genes has emerged to explain the drug-resistant mechanisms of *P. digitatum* [7]. Hamamoto et al. [11] reported that duplication of a 126-bp DNA element in the *cyp51* promoter region led to the increased resistance of *P. digitatum* strains to the antifungal drug imazalil. Another case of imazalil-resistance is associated with up-regulated CYP51 expression caused by the insertion of a 199-bp miniature inverted-repeat transposable element (MITE) in the promoter region [12]. In addition to the overexpression of the *cyp51*, transporter genes from the ABC transporter family and the MFS transporter family have also contributed to fungicide resistance in *P. digitatum.* Two ABC transporter family genes PMR1 and PMR5 have been revealed to mediate DMI fungicide efflux. Further research gave evidence that toxicant efflux system comprised of PMR1 participated directly in the DMI resistance of fungi [13]. Then the genome sequences of three *P. digitatum* strains have become available: the DMI-resistant strain Pd1, Pd01-ZJU and the sensitive strain PHI26 [14, 15]. After that, several ABC transporters were identified from the genome of Pd01-ZJU and seven of them were induced by fungicide imazalil. Besides, a typical MFS member PdMFS1 was characterized to play a role in imazalil-resistance and pathogenicity of *P. digitatum*. Nevertheless, comparative genomics analysis demonstrated that the difference in genome scale was not expectedly distinct between the resistant and susceptible strains, indicating the importance of transcriptional or post-transcriptional regulation in the fungicide resistance [16, 17].

RNA-Seq is one of the most useful next generation sequencing (NGS) methods to fully understand the landscape of a transcriptome. The RNA-Seq data enable us to explain a biology phenomenon or identify relative response genes from the gene-expression perspective and it has already been used in the research of resistant mechanisms of fungi. *Trichophyton rubrum* is the predominant causative agent for superficial dermatomycosis. In order to understand how triazole antifungal agents interact with dermatophytes, the gene expression response of *T. rubrum* to itraconazole was studied by large-scale gene expression profiling. 670 genes have been identified to relate with the response of *T. rubrum* to itraconazole [18]. Another research group evaluated the time-dependent effects of acriflavine on *T. rubrum* transcriptome using the RNA-seq technology. The results provide insights into the molecular events underlying the stress responses of *T. rubrum* to acriflavine [19]. The yeast *Metschnikowia fructicola* is an antagonist with biological control activity against postharvest diseases of several fruits. RNA-Seq technology has been used to check the response of *M. fructicola* to citrus fruit and to the postharvest pathogen, *P. digitatum*, which provided new insight into the biology of the tritrophic interactions in a biocontrol system of the yeast, *M. fructicola* for the control of green mold [20]. A transcriptomic approach has also been applied to investigate the induction of secondary metabolism in citrus fruit in response to *P. digitatum* infection [21]. Many studies have focused on the resistant mechanisms of *P. digitatum* towards antifungal drugs. However, the transcriptomic analysis on the resistant-mechanisms of *P. digitatum* towards prochloraz has been rarely documented. In this study, we provide a whole transcriptome analysis of a prochloraz-resistant strain (HS-F6) and a prochloraz-susceptible strain (HS-E3) before and after prochloraz treatment.

Multidrug efflux transporters have been well-known to play important roles in intrinsic and acquired resistance in many bacteria and in the regulation of fungi resistance to many DMIs drugs. Multidrug efflux transporters, at present, are categorized to five families in terms of their primary structure and particular energy-coupling mode. The MATE (Multidrug and Toxic Compound Extrusion) is one of these families [22]. *NorM* coloned from *Vibrio parahaemolyticus* is the first multidrug transporter categorized in the MATE family. *E.coli* KAM3 cells (lacks AcrB, a component of the AcrAB multidrug transporter, and a restriction system) harboring the plasmids with *NorM* element showed very low levels of norfloxacin accumulation compared to the host cells [23]. In some bacteria, transporter genes of MATE family are connected with resistance to antimicrobial agents and physiological properties of deletion mutants in the MATE-family transporters have been reported. For example, inactivation of *mepA* in multidrug resistant *S. aureus* resulted in decreases in the MICs of several antimicrobial agents, which were indistinguishable from the MIC levels of the original susceptible strain. Besides, the *mepA* gene was shown to be overexpressed in the multidrug resistant *S. aureus* [24].

ATP-Binding cassette transporters (ABC) are the main type of transporters associated with multidrug resistance and have been identified in majority kinds of organisms from bacterial to human. The study in *Candida albicans* has proven that two ABC transporters CDR1 and CDR2 are connected with host resistance to DMIs drugs. Certain important site mutations in *CDR1* and the up-regulation of *CDR1* and *CDR2* significantly increase the resistance of *C. albicans* to DMIs drugs [25]. Two ABC transporter encoding genes *atrA* and *atrB* have been cloned in the filamentous fungi *Aspergillus nidulans*. Research demonstrated that the overexpression of *atrB* led to the resistance of *A. nidulans* to azole drugs [26]. In *P. digitatum*, four ABC transporters PMR1, PMR3, PMR4 and PMR5 have been characterized. Among them, PMR1 and PMR5 are involved in multidrug resistance of *P. digitatum* [13, 27].

MFS (Major Facilitator Super Family) transporters function similar as ABC transporters and they are also ubiquitous in different organisms. CaMDRl is a MFS transporter of *C. albicans* and it played an important role in the resistant mechanisms of the strains to azole drugs [28]. In the fungal wheat pathogen *Mycosphaerella graminicola*, a MFS transporter MgMfsl had been characterized. It reveals that MgMfsl is a strong protectant against natural toxic compounds and fungicides [29]. A MFS transporter PdMFS1 identified in *P. digitatum* is was proved to be partially involved in the imazalil-resistance and pathogenicity [16]. In filamentous fungi, ABC transporters have been widely studied. However, few have focused on the MFS and MATE transporters, which might also play significant roles in fungi resistance.

In this study, the transcriptome of a prochloraz-resistant strain HS-F6 and a prochloraz- susceptible strain HS-E3 had been sequenced and analyzed to figure out the differences of gene expression profiles in the strains with different level of drug resistance. The q-PCR was used to confirm the expression profiles of selected genes in this research.

## Methods

### Strains and media

The *P. digitatum* strain HS-F6 (prochloraz-resistant strain) and HS-E3 (prochloraz- susceptible strain) isolated by our research group [30] were used in this study. HS-F6 is highly resistant to prochloraz with an EC_50_ value of 7.896 mg/L while the EC_50_ of HS-E3 is only 0.01 mg/L. *P. digitatum* strains were cultured on potato dextrose agar (PDA) medium (extract of 200 g potato boiled water, 20 g dextrose, and 15 g agar per liter) at 25 °C.

### RNA preparation and quantitative real-time PCR (q-PCR)

Before RNA extraction, 20 μl of a conidial suspension (10^6^ spores ml^−1^) of *P. digitatum* HS-F6 and HS-E3 strains was cultured in PDB medium at 25 °C for 72 h. Prochloraz-treatment experiment was done for the sample preparation. Prochloraz at the concentration of EC_50_ (7 μg/ml for HS-F6 and 0.01 μg/ml for HS-E3) was added to 100 ml liquid potato dextrose medium with shaking for an extra 6 h after cultured at 25 °C for 48 h. The mycelia were filtered and washed several times using double distilled water. Total RNA was extracted using RNAiso Plus (TaKaRa Biotech. Co., Dalian, China) according to the manufacturer’s protocol. All RNA samples were treated with DNase I (TaKaRa Biotech. Co., Dalian, China) and frozen at −80 °C before transcriptome sequencing. RNA degradation and contamination was monitored on 1 % agarose gels. RNA purity was checked using the NanoPhotometer® spectrophotometer (IMPLEN, CA, USA). RNA concentration was measured using Qubit® RNA Assay Kit in Qubit® 2.0 Flurometer (Life Technologies, CA, USA). RNA integrity was assessed using the RNA Nano 6000 Assay Kit of the Bioanalyzer 2100 system (Agilent Technologies, CA, USA).

First-strand cDNA was prepared by All-in-one First strand cDNA Synthesis Kit (Genecopoeia, Guangzhou, China) following the manufacturer’s protocol. qRT-PCR was performed using a BIO-RAD CFX96 q-PCR system with SYBR Green I fluorescent dye detection. The mRNA abundance was normalized with the housekeeping gene β-actin, and the relative expression levels were calculated using the 2^-ΔΔCt^ method [31].

### Library preparation, clustering and sequencing

A total amount of 3 μg RNA per sample was used as input material for the RNA sample preparations. Sequencing libraries were generated at the Novogene Bioinformatics Institute using NEBNext® Ultra™ RNA Library Prep Kit for Illumina® (NEB, USA) following manufacturer’s recommendations and index codes were added to attribute sequences to each sample. Briefly, mRNA was purified from total RNA using poly-T oligo-attached magnetic beads. Fragmentation was carried out using divalent cations under elevated temperature in NEBNext First Strand Synthesis Reaction Buffer(5X). First strand cDNA was synthesized using random hexamer primer and M-MuLV Reverse Transcriptase(RNase H). Second strand cDNA synthesis was subsequently performed using DNA Polymerase I and RNase H. Remaining overhangs were converted into blunt ends via exonuclease/polymerase activities. After adenylation of 3’ ends of DNA fragments, NEBNext Adaptor with hairpin loop structure were ligated to prepare for hybridization. In order to select cDNA fragments of preferentially 150 ~ 200 bp in length, the library fragments were purified with AMPure XP system (Beckman Coulter, Beverly, USA). Then 3 μl USER Enzyme (NEB, USA) was used with size-selected, adaptor-ligated cDNA at 37 °C for 15 min followed by 5 min at 95 °C before PCR. Then PCR was performed with Phusion High-Fidelity DNA polymerase, Universal PCR primers and Index (X) Primer. At last, PCR products were purified (AMPure XP system) and library quality was assessed on the Agilent Bioanalyzer 2100 system.

The clustering of the index-coded samples was performed on a cBot Cluster Generation System using TruSeq PE Cluster Kit v3-cBot-HS (Illumia) according to the manufacturer’s instructions. After cluster generation, the library preparations were sequenced on an Illumina Hiseq 2000 platform and 100 bp paired-end reads were generated.

### Reads mapping to the reference genome

Raw data (raw reads) of fastq format were firstly processed through in-house perl scripts. In this step, clean data (clean reads) were obtained by removing reads containing adapter, reads containing ploy-N and low quality reads from raw data. At the same time, Q20, Q30 and GC content the clean data were calculated. All the downstream analyses were based on the clean data with high quality.

Reference genome and gene model annotation files were downloaded from genome website (http://genome.jgi.doe.gov/Pendi1/Pendi1.home.html) directly. Index of the reference genome was built using Bowtie v2.0.6 and paired-end clean reads were aligned to the reference genome using TopHat v2.0.9. We selected TopHat as the mapping tool for that TopHat can generate a database of splice junctions based on the gene model annotation file and thus a better mapping result than other non-splice mapping tools.

### Quantification of gene expression level

HTSeq v0.5.4p3 was used to count the reads numbers mapped to each gene. And then RPKM of each gene was calculated based on the length of the gene and reads count mapped to this gene. RPKM, Reads Per Kilobase of exon model per Million mapped reads, considers the effect of sequencing depth and gene length for the reads count at the same time, and is currently the most commonly used method for estimating gene expression levels [32].

### Differential expression analysis

Prior to differential gene expression analysis, for each sequenced library, the read counts were adjusted by edgeR program package through one scaling normalized factor. Differential expression analysis of two conditions was performed using the DEGSeq R package (1.12.0). The P values were adjusted using the Benjamini & Hochberg method. Corrected P-value of 0.005 and log2 (Fold change) of 1 were set as the threshold for significantly differential expression.

Gene Ontology (GO) enrichment analysis of differentially expressed genes was implemented by the GOseq R package, in which gene length bias was corrected. GO terms with corrected Pvalue less than 0.05 were considered significantly enriched by differential expressed genes. KEGG is a database resource for understanding high-level functions and utilities of the biological system, such as the cell, the organism and the ecosystem, from molecular-level information, especially large-scale molecular datasets generated by genome sequencing and other high-through put experimental technologies (http://www.genome.jp/kegg/). We applied KOBAS software to test the statistical enrichment of differential expression genes in KEGG pathways.

### Novel transcripts prediction and alternative splicing analysis

The Cufflinks v2.1.1 Reference Annotation Based Transcript (RABT) assembly method was used to construct and identify both known and novel transcripts from TopHat alignment results. Alternative splicing events were classified to 12 basic types by the software Asprofile v1.0. The number of AS events in each sample was separately estimated.

### SNP analysis

Picard-tools v1.96 and samtools v0.1.18 were used to sort, mark duplicated reads and reorder the bam alignment results of each sample. GATK2 software was used to perform SNP calling.

## Results

### Identification of expressed transcripts in the *P. digitatum* transcriptome

The whole RNA was extracted from a prochloraz-resistant strain HS-F6 and a prochloraz-susceptible strain HS-E3 before and after prochloraz-treatment and sequenced by Illumina technology. The samples were named as following: PdF6-NI (HS-F6 before the prochloraz-treatment); PdF6-MI (HS-F6 after the prochloraz-treatment); PdE3-NI (HS-E3 before the prochloraz-treatment); PdE3-MI (HS-E3 after the prochloraz-treatment). 244, 283, 77 to 28, 124, 623 raw reads were generated for each sample. After quality control, 202, 313, 70 to 236, 628, 24 clean reads and in total 9760 transcripts were obtained from four samples (Additional file 1: Figure S1 and Additional file 2: Table S1). The obtained clean transcripts were used for further analysis. The percentages of genes in different expression levels were listed in Table 1. As can be seen from the table, the distribution of genes of different expression levels in HS-E3 was similar to that of HS-F6 before prochloraz-treatment. However, in the resistant strain HS-F6, the numbers of genes at high expression levels were larger than that in HS-E3 after the treatment, which indicated a response of more genes to prochloraz treatment for the strain HS-F6.

**Table 1:** Percentages of genes in different expression levels

PKM Interval	PdE3_MI	PdE3_NI	PdF6_MI	PdF6_NI
0-1	790(8.10 %)	1010(10.35 %)	492(5.04 %)	641(6.57 %)
1-3	696(7.13 %)	784(8.03 %)	454(4.65 %)	620(6.35 %)
3-15	2058(21.09 %)	2134(21.87 %)	1807(18.52 %)	2264(23.20 %)
15-60	3303(33.85 %)	3042(31.17 %)	3570(36.58 %)	3280(33.61 %)
>60	2912(29.84 %)	2789(28.58 %)	3436(35.21 %)	2954(30.27 %)

In different samples, 91.89 % to 92.89 % of the total reads were mapped to the genome of *P. digitatum*; and 91.23 % to 92.06 % of the reads in each sample were uniquely mapped to the genome. The percentages of reads mapping to the reference genome in different samples were shown in Table 2. Chromosomal distributions of the reads in four samples were shown in Additional file 3: Figure S2. More reads have been identified in the longer chromosomes (Additional file 3: Figure S2).

**Table 2:** Percentages of reads mapping to the reference genome

Sample name	PdE3_MI	PdE3_NI	PdF6_MI	PdF6_NI
Total reads	40462740	44233860	43540794	47325648
Total mapped	37182159 (91.89 %)	40670207 (91.94 %)	40389548 (92.76 %)	44003316 (92.98 %)
Multiple mapped	268916 (0.66 %)	284883 (0.64 %)	517276 (1.19 %)	437468 (0.92 %)
Uniquely mapped	36913243 (91.23 %)	40385324 (91.3 %)	39872272 (91.57 %)	43565848 (92.06 %)
Read-1	18514101 (45.76 %)	20278703 (45.84 %)	19998128 (45.93 %)	21861625 (46.19 %)
Read-2	18399142 (45.47 %)	20106621 (45.46 %)	19874144 (45.64 %)	21704223 (45.86 %)
Reads map to ‘ + ’	18438317(45.57 %)	20164702 (45.59 %)	19923475 (45.76 %)	21777326 (46.02 %)
Reads map to ‘-’	18474926 (45.66 %)	20220622 (45.71 %)	19948797 (45.82 %)	21788522 (46.04 %)
Non-splice reads	30579359 (75.57 %)	33256246 (75.18 %)	33435379 (76.79 %)	36873244 (77.91 %)
Splice reads	6333884 (15.65 %)	7129078 (16.12 %)	6436893 (14.78 %)	6692604 (14.14 %)

### Identification of differentially expressed genes and isoforms between different samples

**Fig. 1:**
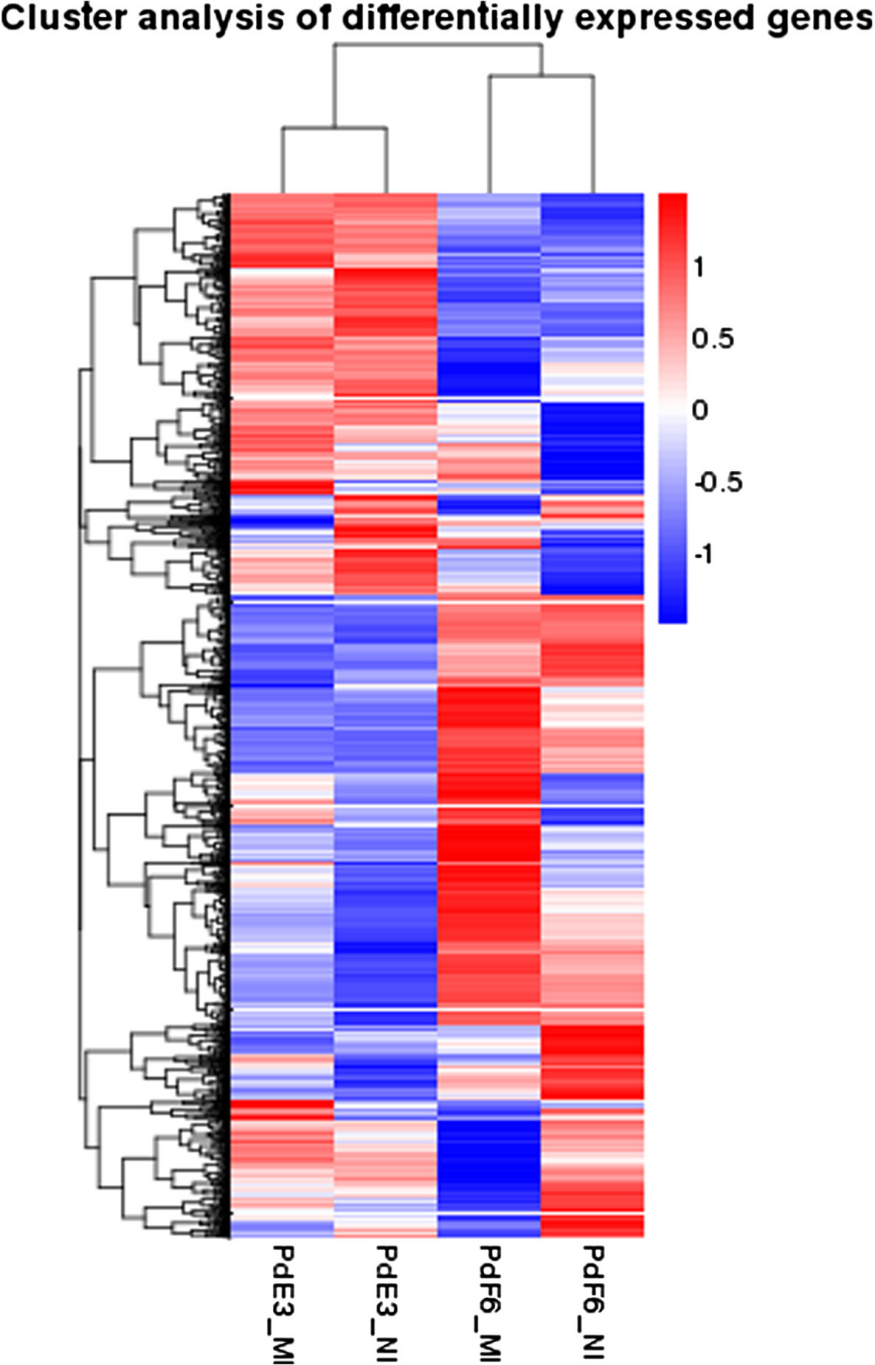
Cluster analysis of differentially expressed genes

To better understand the biological mechanism of prochloraz resistance and drug response, the differentially expressed (DE) genes between different samples were analyzed. The clustering analysis was performed to compare the expression pattern of DE genes in four samples. As shown in Additional file 1: Figure S1, the expression pattern of PdF6_NI and PdF6_MI, PdE3_NI and PdE3_MI were gathering into clusters, which reflected a significant difference in the gene expression between the resistant and susceptible strains. The expression patterns of HS-F6 and HS-E3 before and after the prochloraz-treatment were also different and the difference was more significant in HS-F6, which suggested that a larger number of genes were taking part in the response of HS-F6 towards the drug prochloraz. The dramatic change of the gene expression pattern combined with the DE genes in response to prochloraz conferred the resistance of HS-F6 (Fig. 1).

**Fig. 2:**
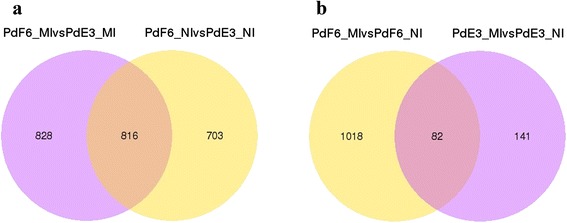
Comparisons of the number and overlapping relationships of DE genes between different samples. **a** Purple circle represents number of DE genes between PdF6_MI and PdE3_MI; yellow circle stand for number of DE genes between PdF6_NI and PdE3_NI. The overlapping region means shared DE genes of two comparable groups. **b** Purple circle represents number of DE genes between PdF6_MI and PdF6_NI; yellow circle stand for number of DE genes between PdE3_MI and PdE3_NI. The overlapping region means shared DE genes of two comparable groups

**Fig. 3:**
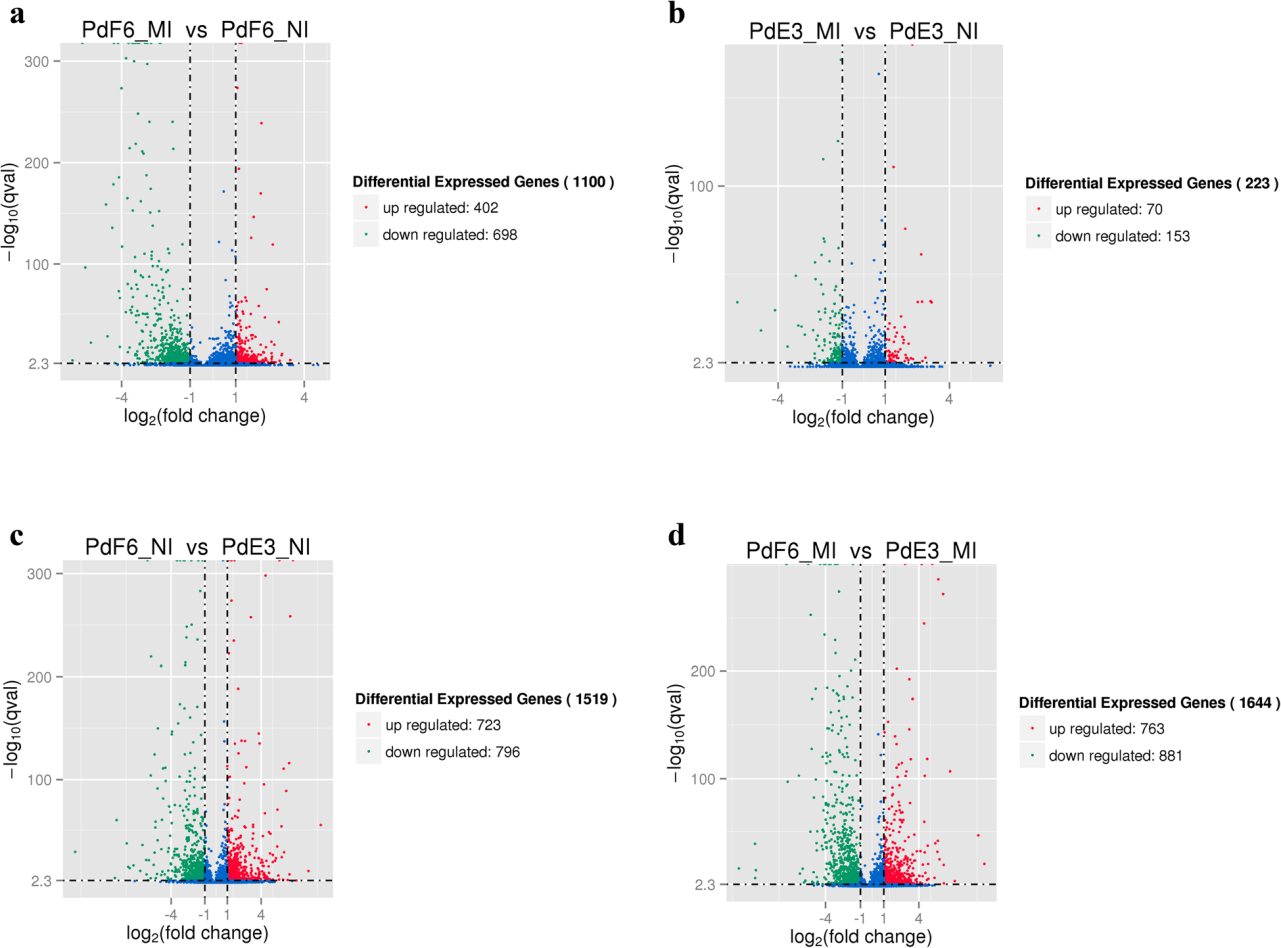
The overall distribution of DE genes in four samples. **a** Comparison group: PdF6_MI and PdF6_NI; **b** Comparison group: PdE3_MI and PdE3_NI; **c** Comparison group: PdF6_NI and PdE3_NI; **d** Comparison group: PdF6_MI and PdE3_MI. The horizontal axis shows the change of expression levels of DE genes in different samples; the vertical axis shows the statistical significance of the change of expression levels. Red dots represent genes which are up regulated and green dots means genes which are down regulated

There were 1519 DE genes between HS-E3 and HS-F6 before prochloraz-treatment when fold changes > =2 and *P* < = 0.05 were used as cutoff values (Fig. 2a), which indicated that the gene expression pattern was changed significantly in the resistant strains. After prochloraz-treatment, there were 223 DE genes in HS-E3 that contained 154 down-regulated and 70 up-regulated DE genes. While in HS-F6, there were 1100 DE genes after prochloraz-treatment, of these 698 were down-regulated and 402 were up-regulated (Fig. 2b). HS-F6 and HS-E3 shared only 82 DE genes after the prochloraz-treatment, and these were important genes involved in the drug response of *P. digitatum*. 1018 DE genes belonged to HS-F6 uniquely were associated with the resistance of HS-F6 to prochloraz. The susceptible strain HS-E3 only had 141 uniquely DE genes. DE genes shared by the two strains and uniquely belonged to HS-F6 after the prochloraz-treatment could be regarded as important genes related to the resistance and would be further studied. The overall distributions of DE genes of different expression levels in four samples were shown in Fig. 3.

**Fig. 4:**
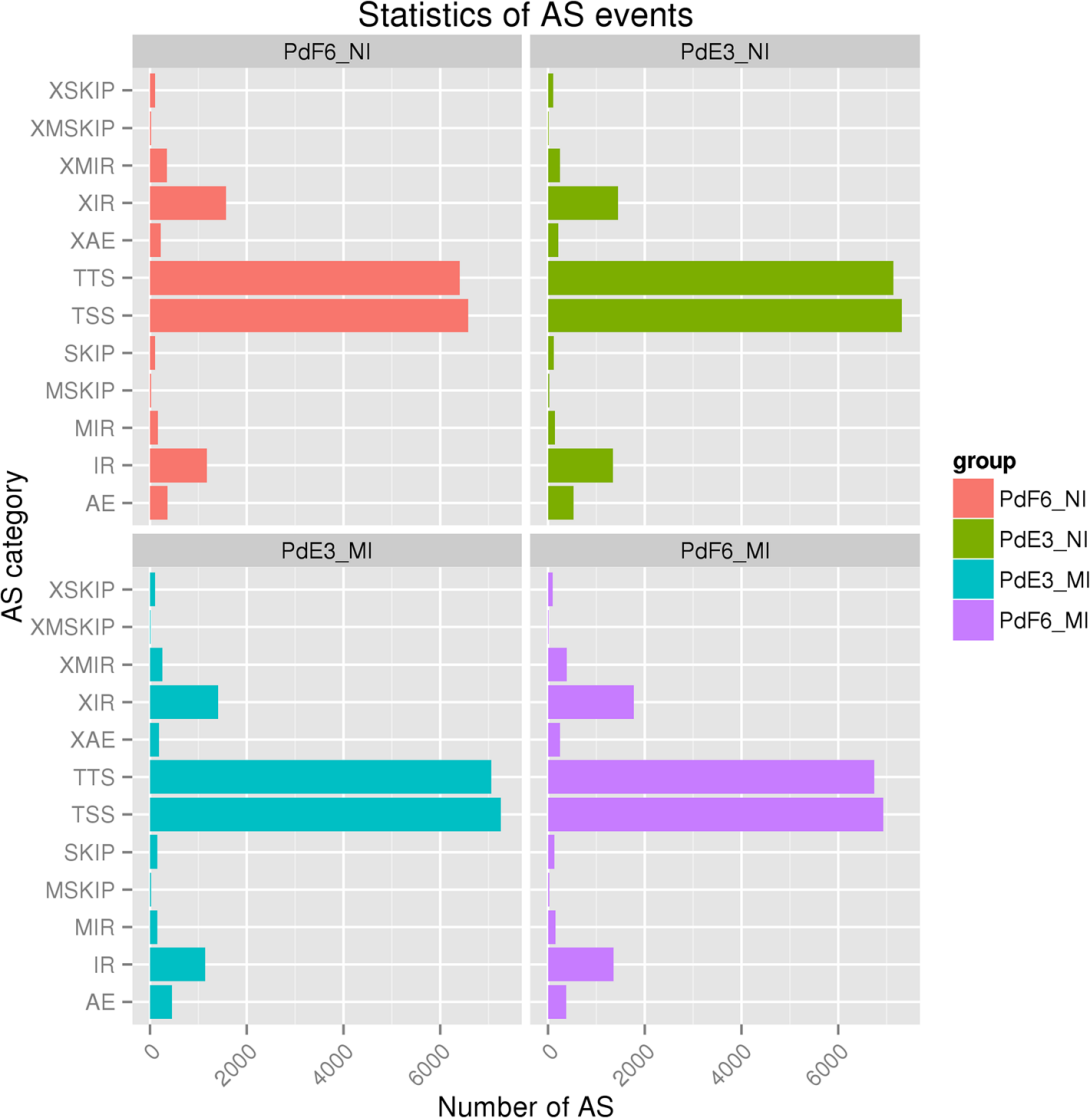
Statistics of splice variants in four samples

Splice variants play an important role in both proteome diversity and specific cellular function. In this study, the chromosomal position of each sample was aligned with the *P. digitatum* genome. Before prochloraz-treatment, 17091 and 18655 splice variants were identified in HS-F6 and HS-E3 respectively. After the treatment, there were 18252 splice variants in HS-F6 and 18198 in HS-E3. Further analysis showed that there were twelve different splice patterns detected in *P. digitatum* transcriptome data, including alternative 5′ first exon (TSS), alternative 3′ last exon (TTS), skipped exon (SKIP), approximate SKIP (XSKIP), multi-exon SKIP (MSKIP), approximate MSKIP (XMSKIP), intron retention (IR), approximate IR (XIR), multi-IR (MIR), approximate MIR (XMIR), alternative exon ends (AE), and approximate AE (XAE). The distribution of different splice variant types in four samples was demonstrated in Fig. 4. TTS and TSS were the major splicing patterns detected in our study, which represented 86 % of the total splicing events; while MXE was a rare event which occurred in only 1.2 % of the total events (Fig. 4).

### Funtional distribution of differentially expressed genes

**Fig. 5:**
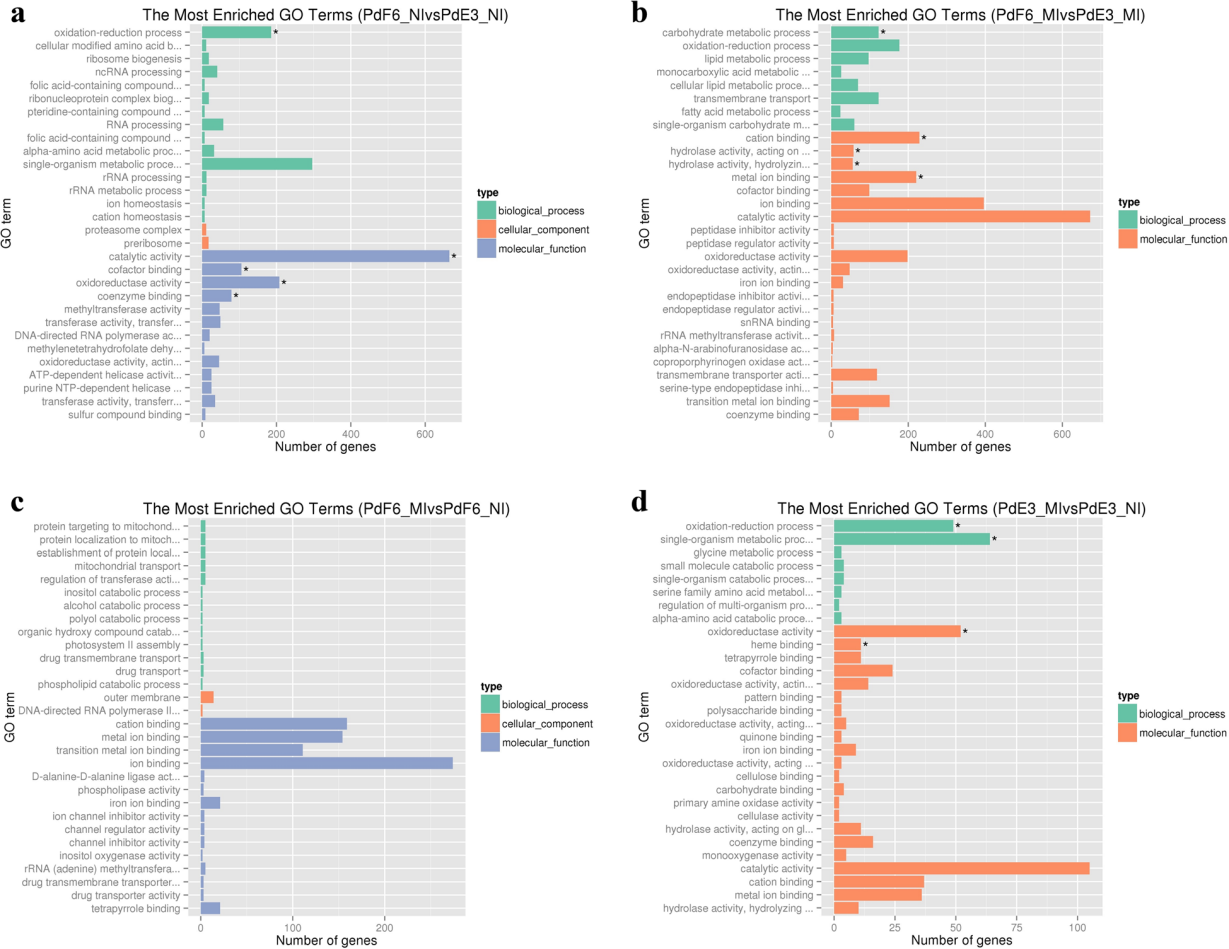
Comparing functional annotations of contigs between different samples. **a** Comparable group: PdF6_NI and PdE3_NI; **b** Comparable group: PdF6_MI and PdE3_MI; **c** Comparable group: PdF6_MI and PdF6_NI; **d** Comparable group: PdE3_MI与PdE3_NI. The green bars represent biological process; orange bars represent cellular component; purple bars represent molecular function

To find out genes related to the high resistance of HS-F6 and responses of *P. digitatum* to antifungal drug prochloraz, differentially expressed genes were further analyzed. The functional distributions of these DE genes were analyzed in four different sample groups (group 1: PdF6_NI and PdE3_NI; group2: PdF6_MI and PdE3_MI; group 3: PdF6_NI and PdF6_MI; group 4: PdE3_MI and PdE3_NI. According to the GO (Gene ontology) categories, in group 1, the 1519 identified DE genes were categorized into three major functional groups: cellular component, molecular function, and biological process. The abundant genes were categorized into 30 major functional groups based on the GO categories; catalytic activity, single − organism metabolic procedure, oxidation − reduction process, oxidoreductase activity and cofactor binding are the top five functional categories (Fig. 5a). In group 2, DE genes in the categories of catalytic activity, ion binding, cation binding, metal ion binding and oxidoreductase activity were the most abundant (Fig. 5b). The DE genes of HS-F6 before and after prochloraz-treatment were also categorized into 30 major functional groups, among them the top five functional categories were ion binding, cation binding, metal ion binding, transition metal ion binding, and tetrapyrrole binding (Fig. 5c). Comparatively, in group 4, top five functional categories are catalytic activity, single − organism metabolic procedure, oxidoreductase activity, oxidation − reduction process, and cation binding (Fig. 5d).

**Fig. 6:**
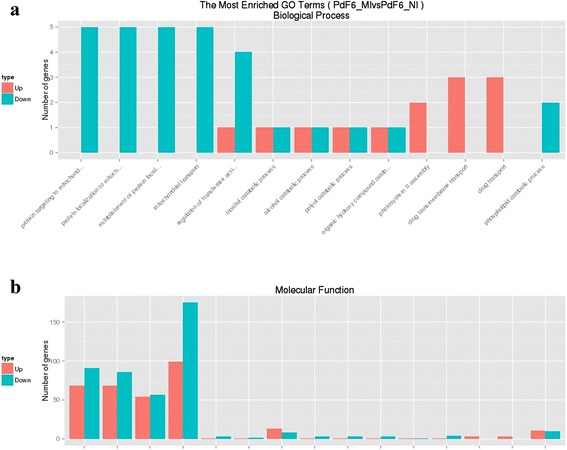
The most enriched GO terms between PdF6_NI and PdF6_MI. **a** Function group: Biological process; **b** Function group: Molecular Function

Notably, in the prochloraz-resistant strain HS-F6, there were three drug transmembrane transporter encoding genes (PDIG_42350m.01, PDIG_35850m.01, PDIG_25390m.01) up-regulated after the prochloraz-treatment (Fig. 6). However, in the prochloraz-susceptible strain HS-E3, the expression levels of these genes did not change after the treatment. Besides, the expression levels of these genes were similar in HS-F6 and HS-E3 before the treatment, which indicated that these transporters were related with prochloraz response and may contribute to the resistance of HS-F6. According to the result of NCBI Blast, these genes were all belonging to the multidrug and toxic compound extrusion family (MATE) which was connected with the extrusion of toxic compounds.

### Real-time PCR validation of differential gene expression of the transporter- encoding genes of *P. digitatum* before and after prochloraz- treatment

**Fig. 7:**
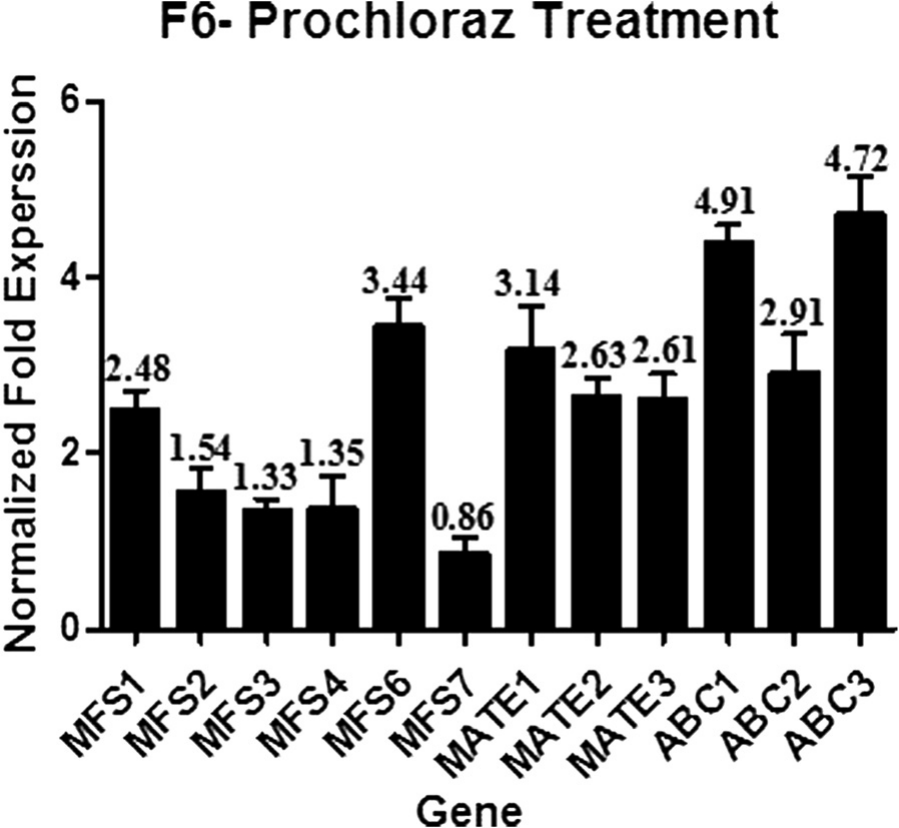
Expression analysis of chosen transporters in HS-F6 after prochloraz-treatment. The mRNA abundance was normalized using the housekeeping gene β-actin, and the relative expression levels were calculated using the 2^-ΔΔCt^ method. Three biological replicates were performed

Analysis and annotation of DE genes revealed that there were a lot of transporter encoding genes up-regulated after the treatment, and some of them were not reported before this study. Real-time PCR was used to validate the expression levels of thirteen transporter genes, including seven MFS (Major Facilitator Superfamily) transporters (PDIG_04260m.01, PDIG_16900m.01, PDIG_51830m.01, PDIG_29550m.01, PDIG_34420m.01, PDIG_69730m.01, PDIG_77390m.01), three ABC (ATP-binding cassette) transporters (PDIG_28350m.01, PDIG_51220m.01, PDIG_66880m.01) and three MATE (multidrug and toxic compound extrusion family) transporters (PDIG_42350m.01, PDIG_35850m.01, PDIG_25390m.01). Among these genes, PDIG_04260m.01 was up-regulated in both HS-F6 and HS-E3 after the prochloraz-treatment. PDIG_16900m.01 and PDIG_29550m.01 were up-regulated in HS-F6 compared with HS-E3 before the treatment. PDIG_16900m.01, PDIG_34420m.01 and PDIG_69730m.01 were up-regulated in HS-F6 after the prochloraz-treatment. PDG_7I7390m.01 was up-regulated in HS-E3 after the prochloraz-treatment. Three ABC transporters and three MATE transporter genes were all up-regulated in HS-F6 but not in HS-E3. The gene expression results from the real-time PCR were consistent with the transcriptome data, most of these drug transporters were up-regulated in HS-F6 after the prochloraz-treatment, thus confirmed the expression pattern of DE genes at four *P. digitatum* samples (Fig. 7). The chosen transporters were listed in Table 3.

**Table 3:** Naming of transporters

Gene ID	Name	Expression pattern
PDIG_04260m.01	MFS-1	Up-regulated in both HS-F6 and HS-E3 after prochloraz-treatment.
PDIG_16900m.01	MFS-2	Up-regulated in HS-F6 compared with HS-E3. Up-regulated in HS-F6 after prochloraz- treatment.
PDIG_29550m.01	MFS-3	Up-regulated in HS-F6 compared with HS-E3.
PDIG_34420m.01	MFS-4	Up-regulated in HS-F6 after prochloraz- treatment.
PDIG_51830m.01	MFS-5	Up-regulated in HS-F6 after prochloraz- treatment.
PDIG_69730m.01	MFS-6	Up-regulated in HS-F6 after prochloraz- treatment.
PDIG_77390m.01	MFS-7	Up-regulated in HS-E3 after prochloraz- treatment.
PDIG_42350m.01	MATE-1	Up-regulated in HS-F6 after prochloraz- treatment.
PDIG_35850m.01	MATE-2	Up-regulated in HS-F6 after prochloraz- treatment.
PDIG_25390m.01	MATE-3	Up-regulated in HS-F6 after prochloraz- treatment.
PDIG_28350m.01	ABC-1	Up-regulated in HS-F6 after prochloraz- treatment.
PDIG_51220m.01	ABC-2	Up-regulated in HS-F6 after prochloraz- treatment.
PDIG_66880m.01	ABC-3	Up-regulated in HS-F6 after prochloraz- treatment.

## Discussion

Fungal disease is one of the major destructive diseases severely affecting the output and quality of crop. Diseases caused by *M. grisea, U. maydis, P. digitatum* and other fungal pathogens led to crop damage, fruit decay and economic loss [33]. Green mold is the most damaging disease of citrus fruit which is caused by *P. digitatum*. According to the data of Chinese Citrus Academic Annual Meeting in 2012, the cultivated area of citrus of China has reaching 3300 million mu and the total output was approximately 2700 million ton (http://www.cqagri.gov.cn/specials/wsgjj/). However, the losses during the postharvest process have been evaluated to be 10 % to 30 % proportion of the total fruit production [34]. Therefore, the controlling of fungal disease has been received more and more attention.

The resistance of fungi was caused by multiple mechanisms including: 1) the mutation of the target protein encoding gene *cyp51*; 2) the over-expression of the target protein; 3) the up-regulation of transporting encoding genes such as *MDR1*,*CDR1* and *CDR2*; 4) the mutation of sterol synthesis genes such as ERG3 which hindered the synthesis of sterol which is an essential component of membrane. The resistance of fungi is closely connected with the target enzyme CYP51. With the widespread using of azole antifungal drugs in agriculture and clinical, the resistance of resistance was even more serious, thus the research of CYP51 target enzyme has gained more attention. In 2014, by cloning the *cyp51A/B/C* genes and their promoters from 78 *P. digitatum* strains of different resistant-levels, our research group analyzed the relationship between the resistance of *P. digitatum* and the mutation of *cyp51A/B/C* genes. It turned out that 4 mutational sites Y136H, Q309H, G459S and F506I in *cyp51B*’s coding areas were closely associated with the resistance of *P.digitatum* [30]. Another resistant-mechanism is the regulation of target protein expression by the transcriptional factors. CaUpc2 is a well-known transcriptional factor that has been proved to be connected with strains sterol metabolism and drug-resistance in *C. albicans*. The expression of sterol synthesis genes *erg2* and *erg11* were regulated by CaUpc2. In *CaUpc2*-disrupted strains, the accumulation of ergosterol was decreasing and the expression of *erg11* was blocked. Therefore, the strain became more susceptible to drugs [35–37].

Except for the target enzyme *cyp51*, transporter genes from ABC, MFS and MATE families are also associated with the resistance of *P. digitatum* to antifungal drugs. One of the resistant-mechanisms of fungal is to reduce the concentration of drug of the mycelium by energy-dependent toxic extrusion which has been reported in several fungi such as *A. nidulans* and *B. cinerea* [38, 39]. A lot of transporter encoding genes were identified from the transcriptome data in this study, including 14 MFS transporters, 8 ABC and 3 MATE transporters. Most of these transporters were up-regulated in HS-F6 or HS-E3 after prochloraz-treatment except for MFS-5. By further analysis, seven MFS transporters (PDIG_ 04260 m.01, PDIG_16900m.01, PDIG_29550m.01, PDIG_34420m.01, PDIG_ 69730 m.01, PDIG_77390m.01), three ABC transporters (PDIG_28350m.01, PDIG_ 51220 m.01, PDIG_66880m.01) and three MATE transporters (PDIG_42350m.01, PDIG_35850m.01, PDIG_25390m.01) were regarded as important transporters connected with drug exclusion (Table 3). Among them, MFS-2 and MFS-3 were up-regulated in HS-F6 compared with HS-E3 before prochloraz-treatment; MFS-1, MFS-2, MFS-4, MFS-5, MFS-6, MATE-1, MATE-2, MATE-3, ABC-1, ABC-2 and ABC-3 were up-regulated in HS-F6 after prochloraz-treatment. MFS-1 and MFS-7 were up-regulated in HS-E3 after prochloraz-treatment. These chosen transporters in the current study have not been investigated before. Besides, most of them were up-regulated in prochloraz-resistant strain HS-F6 before or after prochloraz-treatment, indicating that they might be connected with drug-resistance of HS-F6. Notably, three MATE transporters were identified by Gene ontology (GO) term enrichment of DE genes. They comprised one of the most enrich GO terms (drug trans-membrane transport) of HS-F6 after the prochloraz-treatment, while these gene transcripts were not detected in prochloraz-susceptible strain HS-E3 before and after drug treatment. The result of NCBI Blast analysis revealed that all these genes were the members in MATE family. Drug resistance caused by the extrusion of toxic compounds by transporters from MATE family has already been reported in many kinds of bacterial [24, 25], but it has not been reported in fungi. Thus, the three MATE transporters identified in HS-F6 should be paid more attention in the further study. Further studies are required to explain why these transporters were up-regulated only in the response of HS-F6 to prochloraz and to identify the transporters function involved in the prochloraz resistance. Then real-time PCR was performed to comfirm the expression of these transporters. Except for MFS5 which could not be detected, the expression of the other transporters was all consistent with the transcriptome data (Fig. 7). The results confirmed the expression pattern of DE genes in the four *P. digitatum* samples.

This study researched the resistant mechanisms of *P. digitatum* from the transcriptome perspective for the first time. By comparing the transcriptome data of a prochloraz-susceptible strain HS-E3 and a prochloraz-resistant strain HS-F6 before and after prochloraz-treatment, genes related to drug resistance has been identified. The results revealed that gene expression profile was quite different in two strains before prochloraz-treatment and these DE genes made HS-F6 less susceptible to prochloraz compared with HS-E3. After prochloraz-treatment, a large number of DE genes appeared in HS-F6 and HS-E3 respectively. However, the number of DE genes of HS-F6 was much more than that of HS-E3 which gave the evidence that more genes were required in the response of prochloraz in the resistant strain and the change of expression pattern in HS-F6 was associated with the resistance of the strain to prochloraz. 82 of the DE genes were shared by the two strains. 1018 DE genes were uniquely belonged to HS-F and these genes were regarded as important genes in the study of the resistance of *P. digitatum*. Using GO and KEGG term enrichment combined with local Blast program, 37 important genes were screened from the transcriptome data. These genes composed of novol genes whose expression level changed significantly, cytochrome P450 encoding genes and their putative regulators, MFS transporter encoding genes, ABC transporter encoding genes and MATE transporter encoding genes. The transcriptome data achieved in this study will pave the way for the research of resistance mechanisms of *P. digitatum* towards prochloraz.

## Conclusions

Our results provide the first insight into the resistant mechanisms of *P. digitatum* to antifungal drug prochloraz from the transcriptomic perspective. By comparing the transcriptome data of a prochloraz-resistant strain and a prochloraz-susceptible strain before and after prochloraz-treatment, genes related to prochloraz-response and drug resistance were identified. The expression of 13 transporter encoding genes including 3 MFS transporters, 3 MATE transporters and 7 MFS transporters which are associated with drug-resistant were confirmed by qPCR. 12 of them can be induced by prochloraz in prochloraz-resistant strain, indicating that these transporters are involved in prochloraz-response of *P. digitatum*.

## Availability of supporting data

The datasets supporting the results of this article are available in the NCBI SRA repository [http://www.ncbi.nlm.nih.gov/bioproject/286161].

## Additional file

Additional file 1: Figure S1. Classification of Raw Reads of four samples. (A) Classification of Raw Reads of PdF6_NI; (B) Classification of Raw Reads of PdF6_MI; (C) Classification of Raw Reads of PdE3_NI; (D) Classification of Raw Reads of PdE3_MI. (PNG 1171 kb) Additional file 2: Table S1. Transcriptome data output of four samples. (DOC 36 kb) Additional file 3: Figure S2. Reads density in different chromosomes of four samples. (A) Reads density of PdF6_NI in different chromosomes; (B) Reads density of PdF6_MI in different chromosomes; (C) Reads density of PdE3_NI in different chromosomes; (D) Reads density of PdE3_MI in different chromosomes. (PNG 517 kb)
