# Supplementary figures and images for: Whole transcriptome analysis of Penicillium digitatum strains treatmented with prochloraz reveals their drug-resistant mechanisms
Source: BMC Genomics. 2015 Oct 24;16:855. doi: 10.1186/s12864-015-2043-x (PMC4619488; doi:10.1186/s12864-015-2043-x)

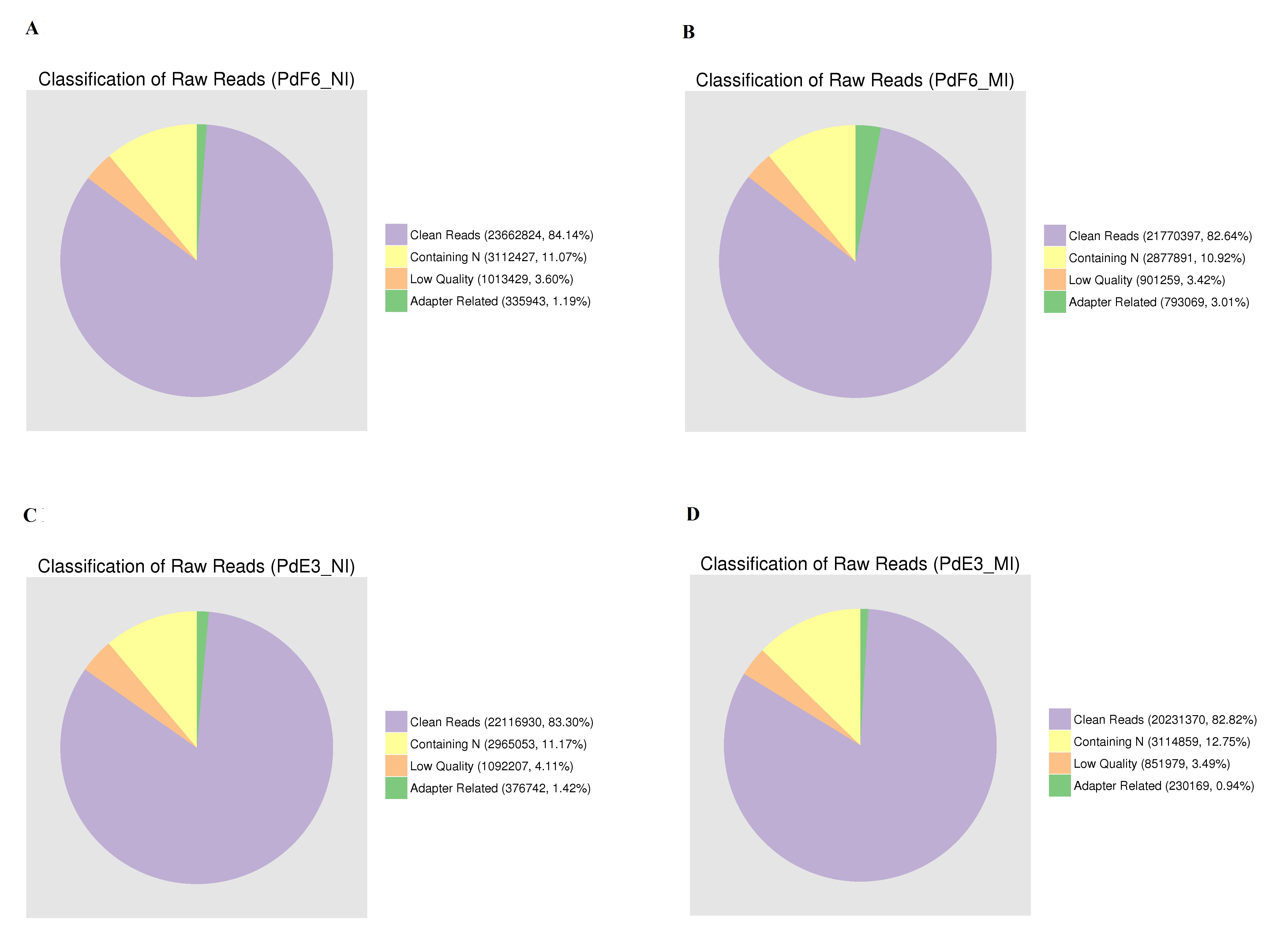

Supplement: Additional file 1: Figure S1. — Classification of Raw Reads of four samples. (A) Classification of Raw Reads of PdF6_NI; (B) Classification of Raw Reads of PdF6_MI; (C) Classification of Raw Reads of PdE3_NI; (D) Classification of Raw Reads of PdE3_MI. (PNG 1171 kb) [file 12864_2015_2043_MOESM1_ESM.png]

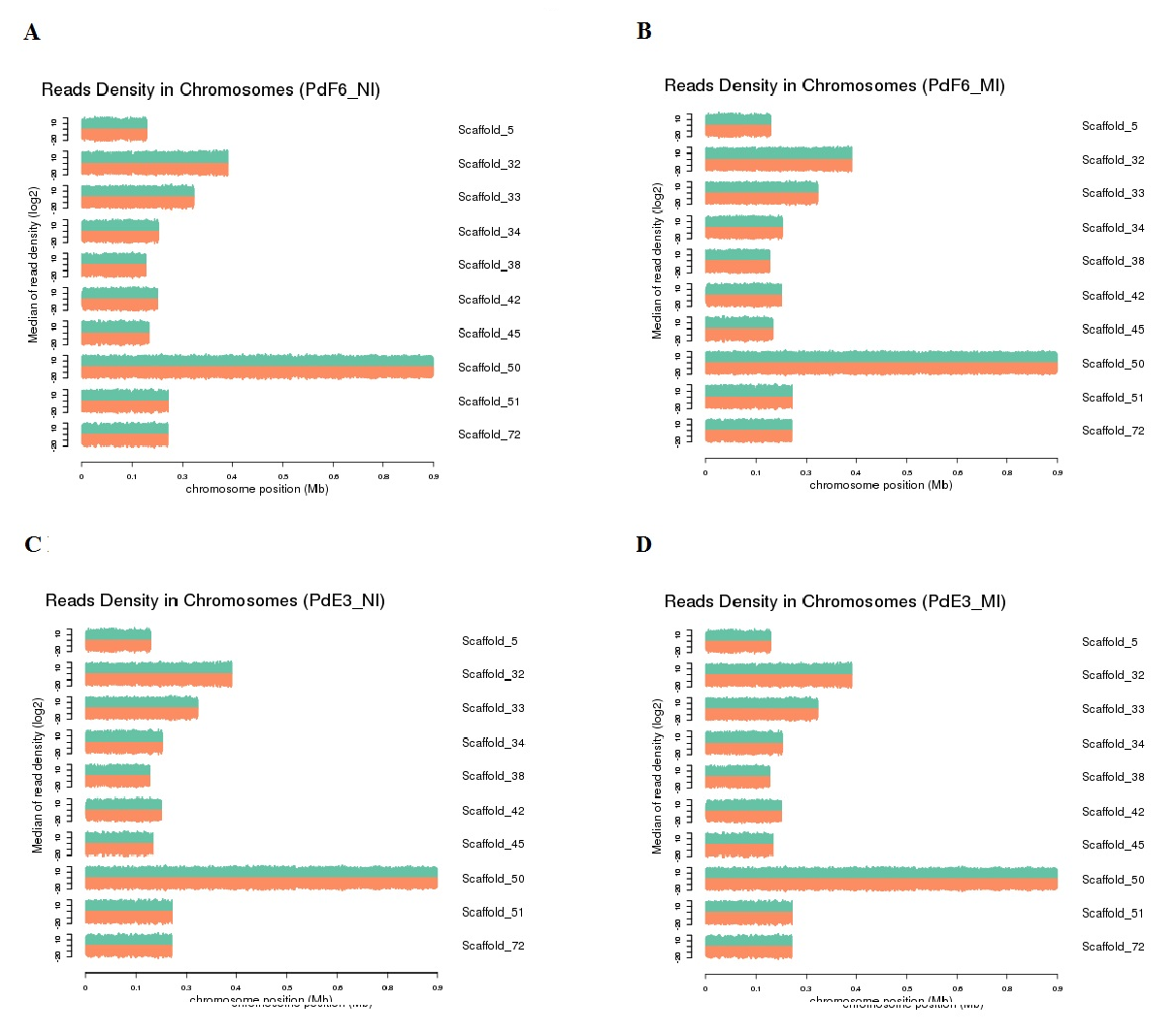

Supplement: Additional file 3: Figure S2. — Reads density in different chromosomes of four samples. (A) Reads density of PdF6_NI in different chromosomes; (B) Reads density of PdF6_MI in different chromosomes; (C) Reads density of PdE3_NI in different chromosomes; (D) Reads density of PdE3_MI in different chromosomes. (PNG 517 kb) [file 12864_2015_2043_MOESM3_ESM.png]
