# Supplementary material for: Whole transcriptome analysis of Penicillium digitatum strains treatmented with prochloraz reveals their drug-resistant mechanisms
Source: BMC Genomics. 2015 Oct 24;16:855. doi: 10.1186/s12864-015-2043-x (PMC4619488; doi:10.1186/s12864-015-2043-x)
Supplement: Additional file 2: Table S1. — Transcriptome data output of four samples. (DOC 36 kb) [file 12864_2015_2043_MOESM2_ESM.doc]

Table S1　Transcriptome data output of four samples

| Sample name | Raw reads | Clean reads | clean bases | Error rate(%) | Q20  (%) | Q30  (%) | Content  (%) |
| --- | --- | --- | --- | --- | --- | --- | --- |
| PdF6_NI_1 | 28124623 | 23662824 | 2.37G | 0.03 | 98.06 | 92.50 | 52.60 |
| PdF6_NI_2 | 28124623 | 23662824 | 2.37G | 0.04 | 96.89 | 90.03 | 52.53 |
| PdF6_MI_1 | 26342616 | 21770397 | 2.18G | 0.03 | 98.04 | 92.52 | 52.17 |
| PdF6_MI_2 | 26342616 | 21770397 | 2.18G | 0.04 | 97.11 | 90.59 | 52.10 |
| PdE3_NI_1 | 26550932 | 22116930 | 2.21G | 0.03 | 98.03 | 92.45 | 52.92 |
| PdE3_NI_2 | 26550932 | 22116930 | 2.21G | 0.04 | 96.73 | 89.67 | 52.86 |
| PdE3_MI_1 | 24428377 | 20231370 | 2.02G | 0.03 | 97.97 | 92.30 | 52.68 |
| PdE3_MI_2 | 24428377 | 20231370 | 2.02G | 0.04 | 97.03 | 90.38 | 52.61 |
